# Mitochondrial Effects of PGC-1alpha Silencing in MPP^+^ Treated Human SH-SY5Y Neuroblastoma Cells

**DOI:** 10.3389/fnmol.2017.00164

**Published:** 2017-05-29

**Authors:** Qinyong Ye, Chun Chen, Erwang Si, Yousheng Cai, Juhua Wang, Wanling Huang, Dongzhu Li, Yingqing Wang, Xiaochun Chen

**Affiliations:** ^1^Department of Neurology, Fujian Institute of Geriatrics, Fujian Medical University Union HospitalFuzhou, China; ^2^Key Laboratory of Brain Aging and Neurodegenerative Diseases, Fujian Key Laboratory of Molecular Neurology, Fujian Medical UniversityFuzhou, China

**Keywords:** Parkinson’s disease, SH-SY5Y cells, PGC-1α, RNA interference, ERRα

## Abstract

The dopaminergic neuron degeneration and loss that occurs in Parkinson’s disease (PD) has been tightly linked to mitochondrial dysfunction. Although the aged-related cause of the mitochondrial defect observed in PD patients remains unclear, nuclear genes are of potential importance to mitochondrial function. Human peroxisome proliferator-activated receptor γ coactivator-1alpha (PGC-1α) is a multi-functional transcription factor that tightly regulates mitochondrial biogenesis and oxidative capacity. The goal of the present study was to explore the potential pathogenic effects of interference by the PGC-1α gene on N-methyl-4-phenylpyridinium ion (MPP^+^)-induced SH-SY5Y cells. We utilized RNA interference (RNAi) technology to probe the pathogenic consequences of inhibiting PGC-1α in the SH-SY5Y cell line. Remarkably, a reduction in PGC-1α resulted in the reduction of mitochondrial membrane potential, intracellular ATP content and intracellular H_2_O_2_ generation, leading to the translocation of cytochrome c (cyt c) to the cytoplasm in the MPP^+^-induced PD cell model. The expression of related proteins in the signaling pathway (e.g., estrogen-related receptor α (ERRα), nuclear respiratory factor 1 (NRF-1), NRF-2 and Peroxisome proliferator-activated receptor γ (PPARγ)) also decreased. Our finding indicates that small interfering RNA (siRNA) interference targeting the PGC-1α gene could inhibit the function of mitochondria in several capacities and that the PGC-1α gene may modulate mitochondrial function by regulating the expression of ERRα, NRF-1, NRF-2 and PPARγ. Thus, PGC-1α can be considered a potential therapeutic target for PD.

## Introduction

Parkinson’s disease (PD) is a neurodegenerative disorder that is characterized by the progressive loss of dopaminergic neurons and affects more than 1% of the population older than 60 years of age (Abou-Sleiman et al., 2006). Both clinical and experimental data have indicated that mitochondrial dysfunction is a common feature of PD that results in a decrease in complex I activity and the overproduction of oxygen radicals (Schapira et al., 1990; Greenamyre et al., 2001). Mitochondria may, therefore, be critical to understanding the etiology of both familial and sporadic PD (Moore et al., 2005; McInnes, 2013). Furthermore, the substantia nigra (SN) of PD patients exhibits a higher mutation rate in mitochondrial DNA than other regions of the brain.

Peroxisome proliferator-activated receptor γ (PPARγ) coactivator 1α (PGC-1α), together with PGC-1β and PGC-related co-activator (PRC), comprise a family of transcriptional co-activators (Scarpulla, 2008a). PGC-1α initiates a diverse set of metabolic programs through its interaction with a variety of transcription factors, including PPARγ (De Nuccio et al., 2011), nuclear respiratory factors 1 and 2 (NRF-1, NRF-2) and estrogen-related receptor α (ERRα; Lin et al., 2005). Additionally, NRF-1, NRF-2, ERRα and PPARγ are primarily responsible for regulating the expression of nuclear-encoded mitochondrial genes, including the components of complexes I-V, cytochrome c (cyt c) and mitochondrial transcription factor A (TFAM; Kelly and Scarpulla, 2004). Furthermore, those nuclear-encoded mitochondrial genes are regulating the energy metabolism of the brain. PGC-1α is, thus, believed to be a major regulator of mammalian mitochondrial biogenesis during physiological or pathological stress.

Recent studies have implicated impaired PGC-1α function in mitochondrial dysfunction in PD. The activation or overexpression of PGC-1α can protect neurons from the loss of mitochondria induced by mutant α-synuclein or mutant huntingtin (Htt; Wareski et al., 2009). Increased PGC-1α expression could improve dopaminergic neuronal viability and mitochondrial activity in *in vivo* and *in vitro* PD models (Mudò et al., 2012; Ferretta et al., 2014; Mäkelä et al., 2016). Our previous work has also suggested that the up-regulation of PGC-1α may have a significant impact on mitochondrial signal transduction by up-regulating the expression of ERRα, NRF-1, NRF-2 and PPARγ (Ye et al., 2016). Meanwhile, PD patients exhibit declining levels of cellular bioenergetic-related gene expression that closely corresponded to the level of PGC-1α (Zheng et al., 2010). However, in the absence of PGC-1α condition, the potential regulation of PGC-1α on mitochondria in *in vitro* PD models is still unclear. Therefore, the down-regulation effect of PGC-1α on related transcription cofactors and mitochondrial function was investigated in PD-liked pathological damage induced by N-methyl-4-phenylpyridinium ion (MPP^+^) in this study.

## Materials and Methods

### Cell Culture

Human SH-SY5Y neuroblastoma cells were obtained from the Chinese Academy of Sciences Committee Type Culture Collection cell bank and were cultured in Dulbecco’s Modified Eagle’s Medium (DMEM/F12, Hyclone, Logan, UT, USA) supplemented with 10% fetal bovine serum (Gibco, Grand Island, NY, USA), 100 U/ml penicillin (Hyclone, Logan, UT, USA) and 100 U/ml streptomycin (Hyclone, Logan, UT, USA; complete media, CM). The cell line was cultured in 100 mm tissue culture plates at 37°C in a humidified incubator (Model No. 3130, Forma Scientific, OH, USA) containing 5% CO_2_. When the cell density reached 80%–90%, the cells were harvested and dispersed. We replaced the culture medium every 2 days. The cells in CM were treated with 1 mM MPP^+^ (D048, Sigma-Aldrich, St. Louis, MO, USA) for 24 h (The antibodies and abbreviations lists see Supplement Materials 1, 2).

### Viral Infection

Human SH-SY5Y neuroblastoma cells were infected through incubation in high titer Adenovirus-Green Fluorescent Protein (Ad-GFP) diluted in a small volume of DMEM/F12 at 37°C for 2 h with gentle swaying every 20 min. The infected cells were maintained for 24 h in fresh CM and were treated with 1 mM MPP^+^ for 24 h. Briefly, 5.0 × 10^3^ cells/well in 100 ul of culture medium were seeded into a 96-well plate and incubated at 37°C in 5% CO_2_ for 24 h to allow cells to grow to 50%–60% confluency. The culture medium was replaced by 100 ul of serum-free medium. The different amount of viruses, 1.25 × 10^5^ pfu/well, 2.5 × 10^5^ pfu/well and 5 × 10^5^ pfu/well, according to multiplicity of infection (MOI; 25, 50, 100) values were applied for infection. The plate was shaken one time every 20 min to increase infection efficiency. After 2 h incubation, the medium was replaced by 100 ul of 5% FBS DMEM/F12 medium. The expression of GFP was observed by fluorescence microscopy (Leica, Germany) 24 h after infection. The transfer efficiency of adenovirus to the SH-SY5Y cells was relatively high, and a MOI of 50 was found to be the most suitable. This MOI was predicted to infect 90%–100% of the SH-SY5Y cells. Then SH-SY5Y cells were also infected with adenovirus in 6-well plates. The number of cells per well was 1.0 × 10^5^ cells/well in 2 ml of culture medium, and the corresponding amount of viruses was 5 × 10^6^ pfu/well (MOI = 50). Adenoviral vector delivery of small interfering RNA (siRNA) targeting PGC-1α (GeneBank accession number: NM_013261) and nonsense control (Ad) were purchased from SBO Medical Biotechnology Co., Ltd (Shanghai, China). The sequences of siRNAs were as follows:

**Table:** 

Marker	Target Sequence	Unit	GC%
siRNA PGC-1	GCAATAAAGCGAAGAGTAT	4.4 × 10^11^ pfu/ml	36.9
siRNA PGC-2	CCACCACTCCTCCTCATAA	4.3 × 10^11^ pfu/ml	52.6
siRNA PGC-3	CCGAAATTCTCCCTTGTAT	1.3 × 10^11^ pfu/ml	42.1
siRNA PGC-4	GCTATGGTTTCATTACCTA	2.1 × 10^11^ pfu/ml	36.9
Ad	TTCTCCGAACGTGTCACGT	1.4 × 10^11^ pfu/ml	52.6

### 3-[4,5-dimethylthiazol-2-yl]-2,5-diphenyl-tetrazolium bromide (MTT) Assay to Evaluate Cell Survival

3-[4,5-dimethylthiazol-2-yl]-2,5-diphenyl-tetrazolium bromide (MTT; Solarbio, Beijing, China) is absorbed into cells and then converted to formazan by mitochondrial succinate dehydrogenase. The accumulation of formazan directly reflects the activity of mitochondria and indirectly reflects cell viability. Cells were plated at a density of 1 × 10^4^ cells/well in 96-well plates and were cultured, differentiated and treated according to the above described methods. Twenty microliter of 0.5 mg/ml MTT was added to each well. After 4 h of incubation at 37°C, the initial 220 μl of solution was removed from each well, and then 100 μl of dimethyl sulfoxide (DMSO) was added to each well. Optical density (OD) was evaluated at 570 nm on an ELISA plate reader (Bio-Rad, Hercules, CA, USA) after the precipitate in the well was dissolved on a microplate mixer for 10 min. All results were normalized against the OD measured in a well under the same conditions without cell culture.

### Detection of Tyrosine Hydroxylase (TH) in SH-SY5Y Cells by Immunocytochemistry

Human SH-SY5Y neuroblastoma cells were permeabilized and fixed with 0.5% Triton X-100 and 4% paraformaldehyde. Slides were blocked with 1% normal donkey serum (Merck, Darmstadt, Germany) in phosphate buffered saline (PBS) for 60 min at room temperature. Cells were washed with 0.1% bovine serum albumin (BSA, Beyotime Institute of Biotechnology, Shanghai, China) in PBS three times with gentle shaking, then incubated with the primary antibody Tyrosine Hydroxylase (TH; 1:100, Santa Cruz, CA, USA) diluted in 0.1% BSA/PBS at 4°C overnight. Labeled donkey anti-rabbit IgG (1:1000 Invitrogen, Paisley, UK) was used as the secondary antibody and was incubated in the dark for 2 h at room temperature. The samples were subjected to chromogenic diaminobenzidine (DAB) staining. In general, one drop of A, B, C reagents was respectively added into 1 ml of distilled water. The mixture was used for cell staining. The color development was monitored under the microscope at room temperature. The reaction stopped by adding distilled water when the ideal color was developed. Hematoxylin was used as a counterstain. The samples mounted by mounting medium were visualized by an inverted microscope under 200 times and 400 times magnification.

### Western Blot Analysis

Human SH-SY5Y neuroblastoma cells, plated at a density of 1 × 10^5^ cells per 6-well dish, were treated according to the aforementioned methods. The cells were washed with ice-cold PBS three times, then the PBS was removed, and the cells were harvested in RIPA Lysis Buffer [50 mM Tris pH 7.4, 150 mM NaCl, 1% Triton X-100, 1% sodium deoxycholate, 0.1% SDS, 1 mM sodium orthovanadate, 50 mM sodium fluoride, 1 mM Ethylenediaminetetraacetic acid (EDTA)], and 0.5 mM phenylmethanesulfonylfluoride (PMSF; Beyotime Institute of Biotechnology, Shanghai, China). The lysates were incubated for 10 min on ice and centrifuged at 12,000× *g* for 10 min at 4°C. The supernatant containing the cell lysates was collected. The protein concentration was measured using BCA Protein Assay Kit (Beyotime Institute of Biotechnology, Shanghai, China). Thirty micrograms of proteins from total cell lysates were denatured by boiling in 1× SDS sample buffer (P0015, Beyotime Institute of Biotechnology, Shanghai, China). Thirty micrograms of denatured protein by boiling were loaded per lane and resolved by 10% sodium dodecyl sulfate-polyacrylamide gel electrophoresis (SDS-PAGE) for 90 min at 80 V. The separated proteins were transferred onto polyvinylidene fluoride (PVDF) membranes (Millipore, Carrigtwohill, Ireland) for 2 h at 200 mA with Bradford reagent (Bio-Rad, Hercules, CA, USA). The membranes were blocked with 5% skim milk in 1× PBS containing 0.05% Tween 20 (PBST) for 4 h at room temperature. The following primary antibodies were incubated with: anti-PGC-1α (1:1500 EMD Millipore Billerica, MA, USA); anti-ERRα (1:2500 EMD Millipore Billerica, MA, USA); anti-NRF1 (1:800 Abcam Cambridge, MA, USA); anti-NRF2 (1:1000 Abcam Cambridge, MA, USA), anti-PPARγ (1:1000 Abcam Cambridge, MA, USA), anti-Actin (1:2000 Beyotime Company of Biotechnology Shanghai, China), anti-GAPDH (1:1000 Beyotime Company of Biotechnology Shanghai, China) in PBST at 4°C overnight, the membranes were washed three times in PBST for 10 min. Subsequently, the membranes were incubated for 1.5 h in PBST containing secondary antibody conjugated to horseradish peroxidase (HRP; anti-mouse IgG 1:2000 and anti-rabbit IgG 1:2000, Beyotime Institute of Biotechnology, Shanghai, China). The immunoreactive bands were visualized and quantified using the enhanced chemiluminescence (ECL) detection kit (Millipore, USA). Protein levels were normalized to the housekeeping protein β-actin or GAPDH to adjust for variability of protein loading and expressed as a percentage of the vehicle control (deemed to be 100%).

### Quantitative Real-Time PCR Analysis

Total RNA from human SH-SY5Y neuroblastoma cells was isolated according to the manufacturer’s protocol using TRizol reagent (Invitrogen, Carlsbad, CA, USA). Total RNA purity and integrity was confirmed using an ND-1000 NanoDrop (NanoDrop Technologies, Wilmington, NC, USA) and 2100 Bioanalyzer (Agilent, Santa Clara, CA, USA). RNA (1 μg) was reverse-transcribed into cDNA in a total volume of 20 μl using the RevertAid^TM^ First Strand cDNA Synthesis Kit (k1621, Fermentas, St. Leon-Rot, Germany). The cDNA (2 μl) was amplified using a sequence detection system (ABI Prism 7500) in a total volume of 20 μl containing 10 μl of Fast Start Universal SYBR Green Master Mix (ROX; Roche, Penzberg, Germany) and each primer at 0.3 μM. Quantitative real-time PCR was performed using the ABI prism 7500 HT sequence detection system (Applied Biosystems, Forster City, CA, USA) based on the 59-nuclease assay for the indicated genes and the housekeeping gene GAPDH. Relative quantification of mRNAs was calculated with the ^∆∆^Ct method. Relative expression was calculated using the ^∆∆^Ct method. The absolute value of the slope of ∆CT vs. a log input <0.1 was considered as a criterion for passing the validation experiment. PCR amplification was carried out on cDNA equivalent to 10 ng of starting mRNA with the following specific oligonucleotide primers:

PGC-1α (Forward, 5-acacagtcgcagtcacaacac-3

Reverse, 5-gcagttccagagagttccaca-3,)

GAPDH (Forward, 5-agaaggctggggctcatttg -3

*Reverse, 5-aggggccatccacagtcttc-3)*.

The conditions for PCR were as follows: initial denaturation for 3 min followed by 40 cycles of two steps: 1st: 95°C for 1 min, 2nd: 72°C (annealing) for 1 min 30 s. Followed by a final incubation of 72°C for 10 min. The results are expressed as the average of triplicate samples from at least three independent experiments for both control and treated cells.

### Mitochondrial Membrane Potential

Loss of mitochondrial membrane potential was assessed using Rhodamine 123 (Sigma, USA). Human SH-SY5Y neuroblastoma cells were seeded in 6-well plates at a concentration of 10^5^ cells per well. After the cells were treated with viral infection and MPP^+^, the medium was removed, and the cells were washed three times with DMEM/F12. The cells were incubated with Rhodamine 123 (Rh123) at a final concentration of 10 μg/ml in FBS-free DMEM/F12 for 30 min at 37°C. The fluorescence signal was measured using a flow cytometer (FACScalibur, San Jose, CA, USA) with excitation and emission wavelengths set at 530 and 590 nm, respectively. For each analysis, 10,000 events were recorded. The value for each treatment group was converted to a percentage of the control value.

### Intracellular ATP Measurement

ATP levels were assessed using a luciferin/luciferase-based ATP assay kit (no. 11699709001, Roche). Briefly, SH-SY5Y cells were treated with 1 mM MPP^+^ for 24 h. Cells were harvested, centrifuged and diluted to a concentration of 1 × 10^6^ cells/ml. The cells were plated at 25 μl/well in black 96-well plates. An ATP standard was serially diluted in dilution buffer to the range of 10^−6^ to 10^−12^ M ATP. The same volume of cell lysis reagent was added to the samples, which were then incubated for 5 min at 25°C. Appropriate volumes of luciferase reagents were added to the samples and readings were recorded between 1 s and 10 s at an interval of 1 s using a multifunctional microplate reader (SpectraMax M3, Sunnyvale, CA, USA).

### Intracellular H_2_O_2_ Measurement

Intracellular production of H_2_O_2_ was fluorometrically assayed according to the manufacturer’s instructions (Amplex® Red, Molecular Probes, A22188, Invitrogen, Eugene, OR, USA). The Amplex® Red reagent reacts with H_2_O_2_ in a 1:1 stoichiometry to produce the red-fluorescent oxidation product, resorufin. The resorufin was measured with excitation and emission wavelengths at 530 and 590 nm. SH-SY5Y cells in which the PGC-1α gene was silenced were treated with 1 mM MPP^+^ for 24 h. Cells were harvested, centrifuged, and diluted to a concentration of 1 × 10^6^ cells/ml. An H_2_O_2_ standard curve was prepared, and the cells were plated on black 96-well plates to be tested. Cultures were exposed to a working solution containing 50 μM Amplex Red reagent and 0.1 U/ml HRP for 30 min and were assayed using a multifunctional microplate reader (SpectraMax M3, Sunnyvale, CA, USA) equipped for excitation at 540 nm and fluorescence emission at 590 nm.

### Intracellular cyt c Measurement by ELISA

Mitochondria were extracted according to the Mitochondria Fractionation Kit (Active Motif, Cat.40015, Carlsbad, CA 92008, USA) instructions. A cyt c standard was serially diluted with dilution buffer to 5.0, 2.5, 1.25, 0.625, 0.31, 0.155, 0.078 and 0 ng/ml according to the instructions accompanying the cyt c Human ELISA Kit (Abcam, Cambridge, MA, USA). The sample and standard specimens were added to 96-well plates filled with equal volumes (100 μl/well), specimen diluent was added to the blank wells, and biotinylated antibody solution (50 μl/well) was incubated for 2 h in the dark at room temperature. Then, the enzyme conjugate working solution was added (100 μl/well) after washing the plates three times for 1 h in the dark at room temperature. Next, the reaction was terminated after the addition of chromogenic substrate for 25 min, and the results were read using a multifunctional microplate reader (SpectraMax M3, Sunnyvale, CA, USA) at OD 450.

### Data Analysis

All quantitative data were collected from at least three independent experiments. The final data are expressed as the mean ± SEM, and analyzed using SPSS 17.0 statistical software (SPSS, Inc., Chicago, IL, USA) by means of one-way analysis of variance (ANOVA), followed by Tukey’s multiple comparison *post hoc* test. Real Time PCR data (Ct) were translated into the 2^−∆∆Ct^ format for statistical analysis; differences between mean values were analyzed by one-way ANOVA, *P* < 0.05 and *P* < 0.01 were considered as significant.

## Results

### Cell Viability after MPP^+^-Induced Toxicity in SH-SY5Y Cells

**Figure 1:**
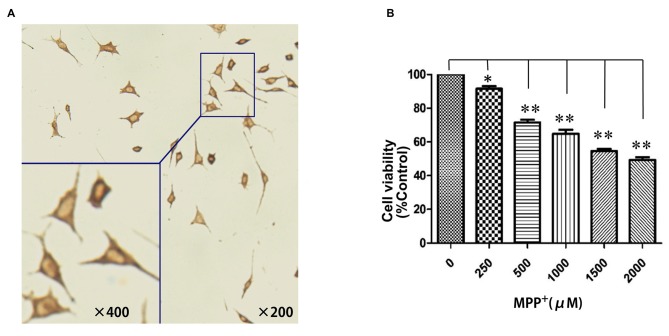
Establishment of a dopaminergic neuronal-like Parkinson’s disease (PD) cell model. **(A)** SH-SY5Y cells showed distinct positive TH staining in the cytoplasm; **(B)** N-methyl-4-phenylpyridinium ion (MPP^+^; 0 μM–2 mM) treatment for 24 h. (*P < 0.05, **P < 0.01, vs. controls. *n* = 4 for cell viability; *n* = 5 for immunocytochemistry staining. Data were analyzed by analysis of variance (ANOVA), followed by Tukey’s LSD *post hoc* tests).

To establish an MPP^+^-induced cell model of PD for investigating the potential pathogenic effects of PGC-1α on PD, we first examined whether SH-SY5Y cell could be eligible for a dopaminergic neuronal-like cell model of PD by expressing TH. As shown in Figure 1A, the brown particles staining in cytoplasm of SH-SY5Y cells but not in nuclei suggested that SH-SY5Y cells could be used as a dopaminergic neuronal-like PD cell model due to their expression of TH. Next, to evaluate the viability of SH-SY5Y cells after exposure to oxidative injury, cells were treated with several concentrations of MPP^+^ (0 μM–2 mM) for 24 h. Cell viability was measured using the MTT assay. MPP^+^ significantly decreased MTT levels, indicating that cell loss is concentration-dependent. In addition, 1 mM MPP^+^ was selected as an optimal concentration when cell viability was decreased by 35.24% (*P* < 0.01; Figure 1B).

### Selection of the Most Efficient siRNA Specific to PGC-1α

**Figure 2:**
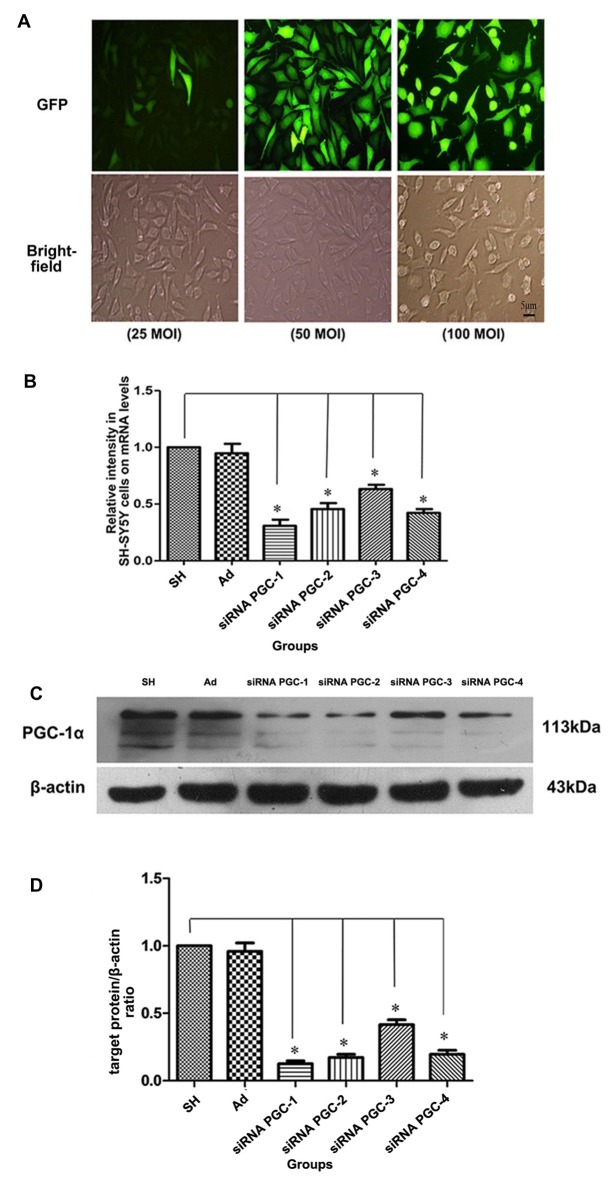
Testing for most efficient infection multiple and silencing conditions of peroxisome proliferator-activated receptor γ coactivator-1alpha (PGC-1α) gene in SH-SY5Y cells. **(A)** On upper panel, fluorescent images from green fluorescent protein (GFP) of SH-SY5Y cells infected with adenovirus vectors (25 multiplicity of infection (MOI), 50 MOI, 100 MOI) for 24 h. On the lower panel, the bright-field images from the same fields as fluorescent ones.** (B)** The relative expression level of PGC-1α mRNA in different groups was assessed by real-time PCR after SH-SY5Y cells not transfected with any viral vector, SH-SY5Y infected with adenoviruses carrying nonsense control, small interfering RNA (siRNA) PGC-1, siRNA PGC-2, siRNA PGC-3 and siRNA PGC-4, respectively.** (C)** Representative image of PGC-1α in the different groups by western blot analysis. The groups: SH (control group, SH-SY5Y cells not transfected with any viral vector group), Ad (nonsense control group), siRNA PGC-1, siRNA PGC-2, siRNA PGC-3 and siRNA PGC-4. **(D)** Quantification of PGC-1α protein in the different groups by Image J. (*P < 0.01 vs. controls. *n* = 4 for fluorescent image; *n* = 5 for real-time PCR; *n* = 8 for western blots. Data were analyzed by ANOVA, followed by Tukey’s LSD *post hoc* tests).

Real-time PCR and Western blot were used to detect the expression of PGC-1α mRNA and PGC-1α protein, respectively, in each group. In order to clarify the infection level of adenovirus to SH-SY5Y cells, SH-SY5Y cells were infected by adenovirus expressing GFP with MOI 25, 50 and 100. The expression level of GFP was observed under fluorescence microscope and the cells were counted (Figure 2A). The results showed that SH-SY5Y cells could be infected by adenovirus efficiently with MOI 50 and 100. However, SH-SY5Y cells infected with MOI 100 showed severe toxicity when comparing to those infected with MOI 50. Therefore, for follow-up experiments we chose MOI 50. The PGC-1α mRNA and protein levels of groups transfected with siRNA PGC-1, siRNA PGC-2, siRNA PGC-3 and siRNA PGC-4 were lower than those of the control group (*P* < 0.01), among which the PGC-1 group was the most marked: gene and protein expression levels were 30.74% (Figure 2B) and 15.56% (Figures 2C,D) of the wild type, respectively. There was no difference between the control group and the nonsense control group. This indicated that PGC-1 was the most efficient siRNA specific to PGC-1α in SH-SY5Y cells.

### The PGC-1α mRNA Expression Levels Upon siRNA Silencing

**Figure 3:**
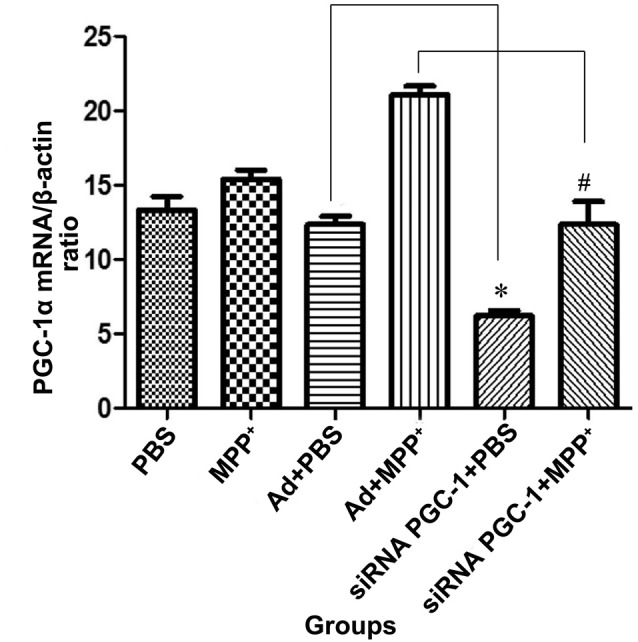
The effect of MPP^+^ and/or siRNA PGC-1 interference on PGC-1α mRNA expression. SH-SY5Y cells treated with MPP^+^ and/or siRNA PGC-1 interference were evaluated for PGC-1α mRNA. The assessed groups were: phosphate buffered saline (PBS; control group), MPP^+^ (1 mM MPP^+^ group), Ad+PBS (negative control group), Ad+MPP^+^ (negative control group with 1 mM MPP^+^), siRNA PGC-1+PBS (PGC-1α gene silencing), siRNA PGC-1+MPP^+^ (PGC-1α gene silencing with 1 mM MPP^+^). (*P < 0.05, siRNA PGC-1+PBS vs. Ad+PBS group; ^#^*P* < 0.05, siRNA PGC-1+MPP^+^ vs. Ad+MPP^+^ group. *n* = 5 for real-time PCR analysis. Data were analyzed by ANOVA, followed by Tukey’s LSD *post hoc* tests).

The real-time PCR showed that (Figure 3) the PGC-1α mRNA level was increased in MPP^+^ treated cells compared with the control group. Compared with the control viral (Ad) group, the expression of PGC-1α mRNA was decreased by 49.67% (*P* < 0.05) in the PGC-1α silencing group. In PD model groups, which further silenced the PGC-1α gene, PGC-1α mRNA expression decreased by 41.34% (*P* < 0.05) compared with the Ad+MPP^+^ groups.

### The Protein Expression Levels of PGC-1α, PPARγ, NRF-1, ERRα and NRF-2 Upon PGC-1α Silencing

**Figure 4:**
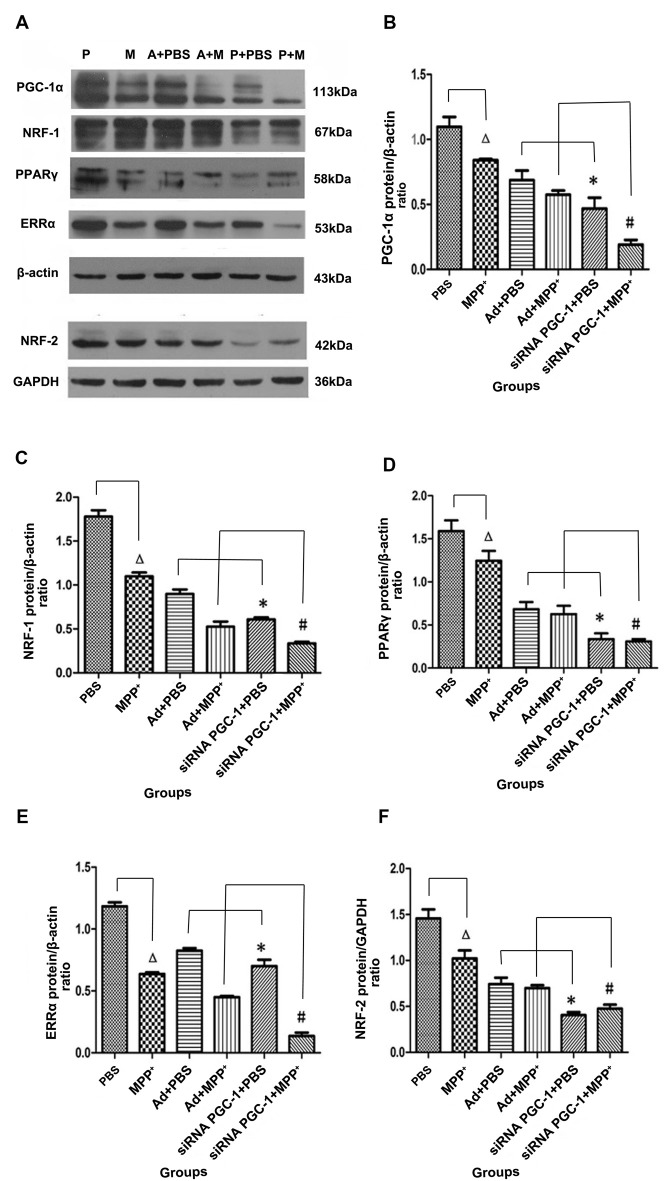
The effects of silencing PGC-1α on the protein levels of its downstream molecules nuclear respiratory factor 1 (NRF-1), peroxisome proliferator-activated receptor γ (PPARγ), estrogen-related receptor α (ERRα), NRF-2 in SH-SY5Y cells treated with MPP^+^ and/or siRNA PGC-1 interference were evaluated for western blot. **(A)** Representative image of western blot analysis of NRF-1, PPARγ, ERRα and NRF-2.** (B–F)** Quantification of PGC-1α, NRF-1, PPARγ, ERRα, NRF-2 protein in different groups by normalizing to β-actin or GAPDH. The groups: PBS (control group), M (MPP^+^ 1 mM group), A (negative control group), P (PGC-1α gene silencing). (^∆^*P* < 0.05, MPP^+^ vs. PBS group; *P < 0.05, siRNA PGC-1+PBS vs. Ad+PBS group; ^**#**^*P* < 0.05, siRNA PGC-1+MPP^+^ vs. Ad+MPP^+^ group; *n* = 10 for western blots. Data were analyzed by ANOVA, followed by Tukey’s LSD *post hoc* tests).

The immunoreactive bands specific to PGC-1α, NRF-1, PPARγ, ERRα, NRF-2, β-actin and GAPDH were present by Western blot (Figure 4A). Compared with the control group, PGC-1α protein expression in the MPP^+^ group was decreased by 23.41% (*P* < 0.05), while PGC-1α protein expression decreased by 57.31% (*P* < 0.01) when the PGC-1α gene was silenced. Compared with the nonsense control group, PGC-1α protein expression in the PGC-1α gene silencing groups decreased by 31.84% (*P* < 0.05). Further treated with MPP^+^ produced a more significant decrease in PGC-1α protein expression of 66.81% (*P* < 0.01), compared with Ad+MPP^+^ group (Figure 4B). As shown in Figure 4C, NRF-1 protein expression decreased by 38.25% (*P* < 0.05) following treatment with MPP^+^ when compared with the nonsense control group, while NRF-1 protein expression decreased by 65.81% (*P* < 0.05) in the PGC-1α gene silencing groups. Compared with the control viral (Ad) groups, NRF-1 protein expression decreased 32.35% (*P* < 0.05) when the PGC-1α gene was silenced. In the PD model groups, upon silencing the PGC-1α gene, NRF-1 protein expression decreased by 69.39% (*P* < 0.05), and compared with the Ad+MPP^+^ groups, NRF-1 protein expression decreased by 36.12% (*P* < 0.05) in the PGC-1α+MPP^+^ groups. At the same time (in Figure 4D), PPARγ protein expression in the MPP^+^ groups decreased by 21.69% (*P* < 0.05) compared with the control group, while PPARγ protein expression decreased more significantly, reaching 78.87% (*P* < 0.05) when the PGC-1α gene was silenced. In PD model groups, in which the PGC-1α gene was further silenced, PPARγ protein expression decreased by 75.32% (*P* < 0.05); compared with the control viral group, PPARγ protein expression in the PGC-1α gene silencing groups decreased by 50.87% (*P* < 0.05). As for ERRα protein (in Figure 4E), compared with the control group, ERRα protein expression decreased by 46.17% (*P* < 0.05) when treated with MPP^+^ only, while ERRα protein expression decreased by 40.74% (*P* < 0.01) in the PGC-1α gene silencing groups. Compared with the nonsense control group, ERRα protein expression decreased 15.10% (*P* < 0.05) when silencing the PGC-1α gene. In PD model groups, which further silenced the PGC-1α gene, ERRα protein expression decreased by 78.66% (*P* < 0.01); compared with the Ad+MPP^+^ groups, ERRα protein expression decreased by 36.12% (*P* < 0.05) in the siRNA PGC-1+MPP^+^ groups. As shown in Figure 4F, NRF-2 protein expression in the MPP^+^ groups decreased by 30.02% (*P* < 0.05) compared with the control group, while NRF-2 protein expression decreased by 73.31% (*P* < 0.01) when silencing the PGC-1α gene. Compared with the control viral (Ad) groups, NRF-2 protein expression decreased by 45.47% (*P* < 0.05) when silencing the PGC-1α gene. Compared with the only MPP^+^ group, which further silenced the PGC-1α gene, NRF-2 protein expression decreased by 55.20% (*P* < 0.05); compared with the control viral group, NRF-2 protein expression in the PGC-1α gene silencing groups decreased by 31.87% (*P* < 0.05).

### Effect of Silencing the PGC-1α Gene on Mitochondrial Function

**Figure 5:**
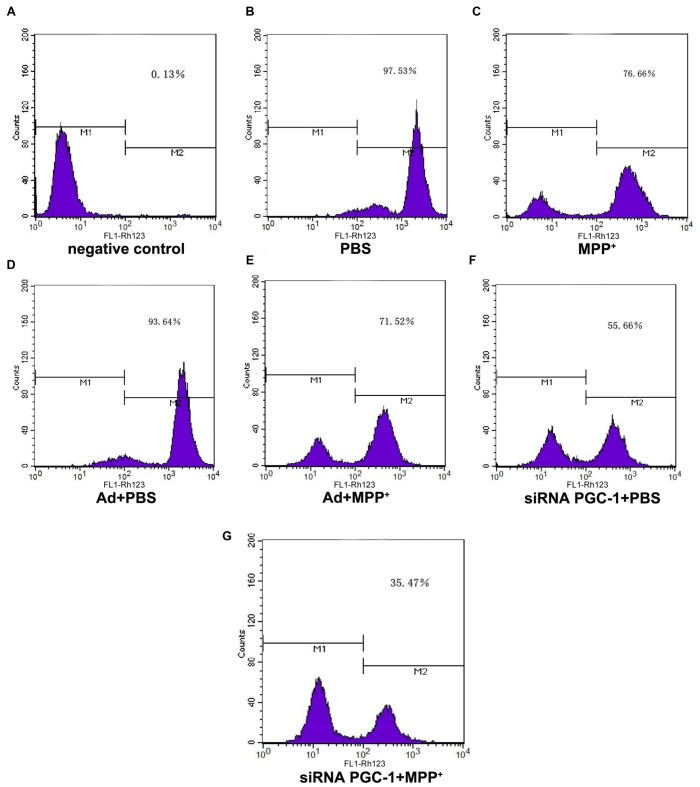
The effect of PGC1-α silencing on mitochondrial membrane potential upon MPP^+^ insult. SH-SY5Y cells treated with MPP^+^ and/or siRNA PGC-1 interference were evaluated for mitochondrial membrane potential. The assessed groups were:** (A)** negative control without Rh123; **(B)** PBS; **(C)** MPP^+^ 1 mM; **(D)** Ad+PBS; **(E)** Ad+MPP^+^ 1 mM; **(F)** siRNA PGC-1+PBS; and **(G)** siRNA PGC-1+MPP^+^ 1 mM. M1: negative control peak; M2: DCFH positive peak; mitochondrial membrane potential was determined by Rh123 fluorescence; *n* = 6 for fluorescence signal measurement.

**Figure 6:**
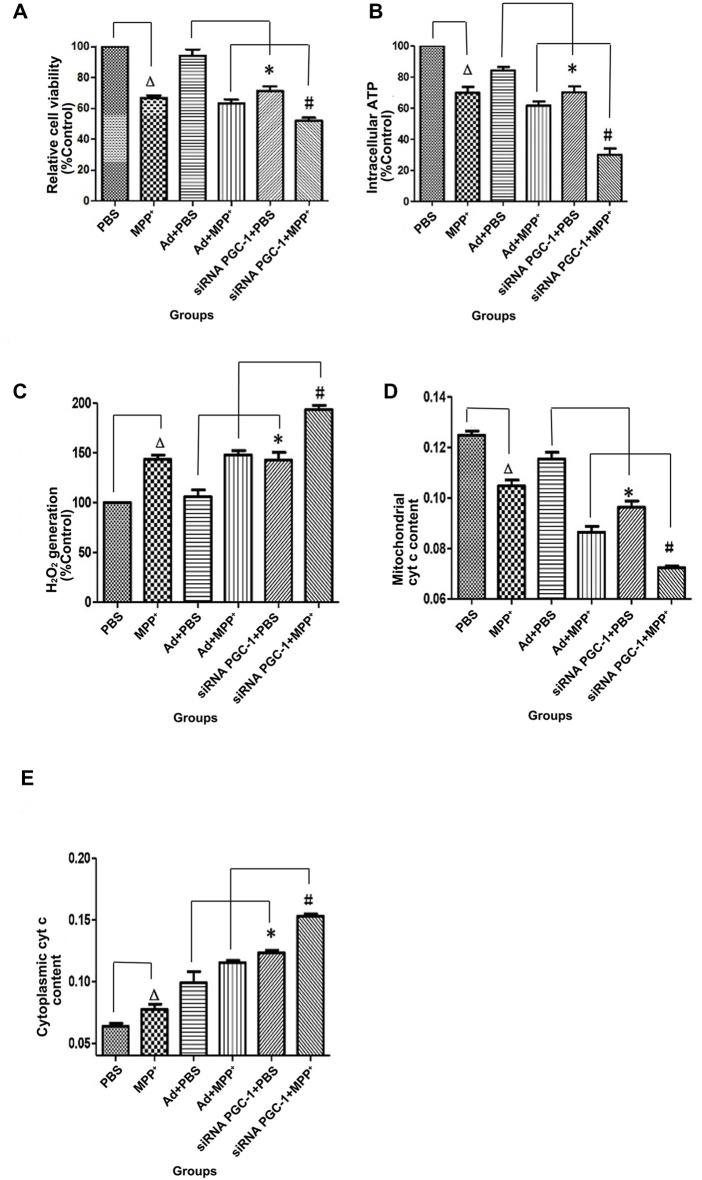
The effects of PGC-1α silencing in SH-SY5Y cells on cell viability, ATP level, H_2_O_2_ level and cytochrome c (cyt c) content upon MPP^+^ insult. SH-SY5Y cells treated with MPP^+^ and/or siRNA PGC-1 interference were evaluated for cell viability, ATP level, H_2_O_2_ level and cyt c content. The assessed groups were: PBS, control group; MPP^+^, MPP^+^ (1 mM); Ad+PBS, nonsense control+PBS; Ad+MPP^+^, nonsense control+1 mM MPP^+^; siRNA PGC-1+PBS, PGC-1α gene silencing, siRNA PGC-1+1 mM MPP^+^, PGC-1α gene silencing+1 mM MPP^+^.** (A)** 3-[4,5-dimethylthiazol-2-yl]-2,5-diphenyl-tetrazolium bromide (MTT) for cell viability. **(B)** Intracellular ATP content was assayed using the luciferase-luciferin reaction. **(C)** Intracellular H_2_O_2_ level was assayed using Amplex® Red reagent. **(D)** Changes in mitochondrial cyt c content.** (E)** Changes of cytoplasmic cyt c content. (^∆^*P* < 0.05, MPP^+^ vs. PBS group; *P < 0.05, siRNA PGC-1+PBS vs. Ad+PBS group; ^#^*P* < 0.05, siRNA PGC-1+MPP^+^ vs. Ad+MPP^+^ group. *n* = 6 for MTT Assay; *n* = 5 for intracellular ATP measurement; *n* = 6 for fluorescence signal measurement by Amplex® Red reagent; *n* = 9 for mitochondrial cyt c measurement by ELISA; *n* = 5 for cytoplasmic cyt c measurement by ELISA. Data were analyzed by ANOVA, followed by Tukey’s LSD *post hoc* tests).

PGC-1α expression is associated with mitochondrial respiration in regulation of energy metabolism and oxidative stress. To analyze the effects of silencing PGC-1α genes on the mitochondrial function of SH-SY5Y cells, we first evaluated whether silencing PGC-1α would affect the mitochondrial membrane potential (∆ψM) using Rh123 as an indicator, as well as cell viability (Figures 5, 6A). Upon MPP^+^ exposure, SH-SY5Y cells showed a statistically significant decrease in ∆ψM and cell viability when compared to the control. However, pre-treatment with PGC-1α silencing further decreased 41.19% (*P* < 0.01) of ∆ψM and 47.15% (*P* < 0.05) of cell viability when compared to SH-SY5Y cells treated with MPP^+^ alone, indicating that expression of PGC-1α rendered dysfuction of mitochondrial respiration induced by MPP^+^ through inhibiting mitochondrial complex I. We then asked whether knockdown of PGC-1α would result in further depletion of ATP upon MPP^+^ treatment. To this end, we measured ATP levels. As shown in Figure 6B, ATP generation in SH-SY5Y cells treated with MPP^+^ was decreased when compared to the control group. However, silencing the PGC-1α gene resulted in a further decrease (63.94%, *P* < 0.05) in ATP level when compared to SH-SY5Y cells treated with MPP^+^ alone. In agreement with the data from mitochondrial potential assay, decreases in ATP levels were correlated with the change in ∆ψM. In fact, decrease of ATP and change of mitochondrial ∆ψM can lead to an increase of reactive oxygen species (ROS). Given that mitochondria generate and accumulate the majority of ROS and H_2_O_2_ and that mitochondrial dysfunction causes an increase in radical production, we next assessed the effect of MPP^+^ on H_2_O_2_ production. As shown in Figure 6C, H_2_O_2_ was significantly increased (43.38%, *P* < 0.01) in SH-SY5Y cells exposed to MPP^+^. However, pre-treatment to silence the PGC-1α gene resulted in a more marked increase H_2_O_2_ level (93.44%, *P* < 0.01) compared to SH-SY5Y cells treated with MPP^+^ alone. In general, overloading of ROS causes cells undergoing apoptosis. We, therefore, detected changes in mitochondrial cyt c using ELISA (Figures 6D,E). Cyt c is an important component of the mitochondrial electron transport chain and is located in the mitochondria, but it can be released into the cytoplasm upon apoptosis. We found that the mitochondrial cyt c level was significantly decreased after SH-SY5Y cells were treated with MPP^+^, and it was reduced further (11.35%, *P* < 0.01) following PGC-1α gene silencing (Figure 6D). Correspondingly, the cyt c in the cytoplasm tended to increase (Figure 6E). In comparison with the control group, cyt c in the mitochondria decreased by 16.02% (*P* < 0.05) after cells were treated with MPP^+^ only. In PD model groups, compared with the control viral groups, the mitochondrial cyt c decreased by 16.22% (*P* < 0.05) in PGC-1α gene silencing groups, and the difference was statistically significant. Meanwhile, cyt c in the cytoplasm increased by 32.66% (*P* < 0.05). Thus, we can conclude that silencing the PGC-1α gene caused mitochondrial damage in the PD cell model and led to mitochondrial cyt c release into the cytoplasm.

## Discussion

PD is a common neurodegenerative disease with clinical features including resting tremor, bradykinesia, rigidity and abnormal posture and gait. In recent decades, the incidence of PD among the aging population in China has increased. This progressive and highly disabling movement disorder has devastating long-term effects for patients, their families and society. For years, many resources have been devoted to exploring the pathogenetic mechanism of PD. More recently, mitochondrial dysfunction and oxidative stress have been widely believed to contribute to the occurrence and development of PD. In this *in vitro* study, we utilized the active ion form (MPP^+^) of the neurotoxin 1-methyl-4-phenyl-1,2,3,6-tetrahydropyridine (MPTP) to establish a cell model of classic PD (Swerdlow et al., 1996). SH-SY5Y cells were fully exposed to a range of MPP^+^ concentrations, and their survival rate decreased in a dose-dependent manner. We found that at 1 mM, MPP^+^ reduced the cell population by nearly one third, and we chose this concentration for our model.

PGC-1α was recently identified as a multifunctional transcriptional coactivator that could regulate mitochondrial biogenesis, oxidative stress, cell metabolism and glucose metabolism through its interaction with relative nuclear receptors (NR). Studies have revealed possible connections between PGC-1α and several neurodegenerative diseases (Rona-Voros and Weydt, 2010; Tsunemi and La Spada, 2012), including Huntington’s disease (HD; Cui et al., 2006), amyotrophic lateral sclerosis (ALS; Zhao et al., 2011), Alzheimer’s disease (AD; Sheng et al., 2012) and PD (Shin et al., 2011). Because no effective inhibitor for PGC-1α exists, we designed an adenovirus vector-based siRNA to target the PGC-1α gene. Our study found that upon suppressing the PGC-1α gene, changes in cellular morphology changed were significantly observed when compared with the controlled groups with respect to PGC-1α knockout mice that have been shown to develop remarkable neuron degeneration in several brain regions, especially the striatum, which is accompanied by abnormal exercise behavior (Lin et al., 2005). Furthermore, with continued MPP^+^ treatment, more cells were lost, which strongly supports the conclusion that PGC-1α knockout mice are highly susceptible to oxidative stress and the neurotoxin MPTP (St-Pierre et al., 2006).

Cyt c is a water-soluble electron carrier located in the intermembrane space (IMS) of mitochondria. It transports electrons from cyt c oxidoreductase (complex III, or cytochrome bc1) to cyt c oxidase (COX, complex IV, or cytochrome a/a3; San Francisco et al., 2013) and can significantly suppress the generation of H_2_O_2_. Mature cyt c is localized to the lateral region of the inner mitochondrial membrane through strong electrostatic and hydrophobic interactions with cardiolipin (Hong et al., 2012). When it is released from the mitochondria into the cytoplasm, cyt c-mediated cell death pathways are activated (Bergstrom et al., 2013). In the cytoplasm, cyt c binds Apaf-1 to form the apoptosome and activate the caspase9 (Li et al., 1997), which functions as an upstream initiator of apoptosis. The caspase pathway may play an important role in the occurrence of PD (Jiang and Wang, 2004). Lin et al. (2005) found that the expression of cyt c in the hearts and brains of PGC-1α knockout mice was decreased compared to wild-type (WT) mice. In our study, mitochondrial cyt c in PGC-1α-silenced cells was also reduced compared to the control group. After MPP^+^ treatment, the level of mitochondrial cyt c decreased further. The level of cytoplasmic cyt c was correspondingly increased. This suggests that the downregulation of PGC-1α could lead to the partial release of cyt c from the mitochondria into the cytoplasm.

PGC-1α is another key transcription cofactor in the oxidative defense system. PGC-1α is co-induced with several ROS-detoxifying enzymes, including copper/zinc superoxide dismutase (SOD1), manganese SOD (SOD 2), catalase, and glutathione peroxidase1 (GPx1). In PGC-1α knockout mice, these enzymes were markedly decreased compared with WT mice (Lin et al., 2005). Thus, the decrease in ROS-detoxifying enzymes could be due to the down-regulation of PGC-1α, which would result in an increased ROS level and the release of cyt c by changing the permeability of the mitochondrial membrane and would eventually induce apoptosis. We also found that when the PGC-1α mRNA was down-regulated/silenced, the stability of mitochondria in both normal and disease model cells was compromised, as was their membrane potential. Because the major functions of mitochondria are mainly related to oxidative phosphorylation and ATP biogenesis, we examined changes in H_2_O_2_ and ATP production and found that the suppression of the PGC-1α gene in normal cells lead to an increase of H_2_O_2_ and a decrease of ATP. These trends were most obvious in the PD model, where apoptosis was observed. We, therefore, concluded that the down-regulation of PGC-1α might not only induce the decrease in mitochondrial membrane potential and ATP production as well as the accumulation of H_2_O_2_, but could also lead to excessive oxidative stress and the loss of dopamine neurons, eventually aggravating PD pathology.

To explore which co-factors are involved in mitochondrial signal transduction, we examined the protein levels of PGC-1α, ERRα, NRF-1, NRF-2, and PPARγ. We found that PGC-1α mRNA in SH-SY5Y cells was increased after MPP^+^ treatment, which could be a short-term stress reaction in response to MPP^+^. The protein levels of PGC-1α, ERRα, NRF-1, NRF-2, and PPARγ consistently decreased after MPP^+^ intervention, indicating that they were sensitive to MPP^+^ toxicity. In the PGC-1α down-regulation group, the levels of ERRα, NRF-1, NRF-2 and PPARγ protein decreased correspondingly.

Similar to PGC-1α, ERRα is mainly expressed in hypermetabolic tissues, including heart, kidney, intestine, skeletal muscle, brown adipose tissue and brain-especially in the hypothalamus (Giguère, 2008). It also regulates mitochondrial biogenesis and oxidative phosphorylation by activating transcription-related genes (Duellman et al., 2010). Cold, physical exercise and hunger stimulate the expression of the PGC-1α and ERRα genes (Cartoni et al., 2005). Both genes are able to regulate the expression of genes related to myocardial metabolism, further promoting the expression of transcription factors and genes that could trans-activate the oxidation of fatty acid. Study found that one surface of the PGC-1α protein reacts specifically with ERRα, and ERRα could in turn regulate the expression of PGC-1α by binding conserved elements in the PGC-1α promoter in cardiomyocytes, suggesting that the interaction between PGC-1α and ERRα is crucial and unique (Schreiber et al., 2003). ERRα exhibited the more obvious response to the suppression of the PGC-1α gene. Thus, we believe that PGC-1α/ERR may play a prominent role in the mitochondrial function of dopaminergic neurons in PD pathogenesis due to their physiological and structural features, although the exact mechanism must be elucidated in future studies.

Nuclear related factors, specifically NRF-1 and NRF-2, are the downstream targets of PGC-1α and key transcription factors involved in mitochondrial biogenesis. Thus far, PGC-1 is thought to be the primary regulator of NRFs (Virbasius and Scarpulla, 1994; Scarpulla, 2008a). Studies have found that both the gene activation and transcription of NRF-1 are induced by the phosphorylation and overexpression of PGC-1α, which in turn modulates the mitochondrial respiratory chain (Yan, 2009; Schilling and Kelly, 2011). The levels of NRF-1 and TFAM in the SN and the striatum decreased in the MPTP-induced PD mice model, whereas the overexpression of NRF-1 or TFAM reversed MPP^+^-induced mitochondrial dysfunction, including the activity of mitochondrial complex I, the mitochondrial membrane potential, and the levels of ATP and ROS (Piao et al., 2012). One study also showed that the mRNA levels of PGC-1α and NRF-1 in the SN and striatum were decreased in a PD mouse model, and there exists a positive correlation between the expression of PGC-1α and NRF-1 (Shin et al., 2011). The expression of NRF-1 and NRF-2 can be increased by PGC-1α in response to the oxidative stress induced by lipopolysaccharide (LPS; Suliman et al., 2004). Our results showed that NRF-1 protein expression was decreased in the PGC-1α silencing group and was accompanied by a decline in the PGC-1α level. Both proteins are significantly depressed by MPP^+^, demonstrating that PGC-1α could also positively regulate NRF-1. The pattern of NRF-2 distribution in the primate visual cortex is virtually identical to that of COX, and both proteins are extremely abundant in cells exhibiting high COX activity (Wong-Riley et al., 2005). Under physiological conditions, NRF-2 is retained in the cytoplasm by the inhibitory protein kelch-like ECH-associated protein 1 (Keap1). Oxidative stress activates NRF-2 and dissociates it from Keap1. NRF-2 translocates into the nucleus and binds to electrophile response element (EpRE) sites to trigger the expression of cytoprotective genes (Bryan et al., 2013). Studies have implicated the transactivational activity of NRF-2 in the expression of respiratory chain enzymes, especially that of some nuclear-encoded COX subunits and human COX VIaL (Ongwijitwat and Wong-Riley, 2004). Moreover, NRF-2 could regulate genes that encode the mitochondrial transcription factors A and B (TFAM, TFB1M and TFB2M). Thus, NRF-2 has the potential to connect the regulation of the nucleus and mitochondria through COX-related gene expression by these two genomes (Scarpulla, 2008b). In our study, NRF-2 protein expression was significantly decreased in the PGC-1α gene silencing group, further suggesting the involvement of NRF-2 in the regulation of PGC-1α by downstream members.

Of note, we previously found that expression of PGC-1α protects mitochondrial dysfunction via increasing mitochondrial membrane potential, reducing the release of mitochondrial cyt c, inhibiting H_2_O_2_ production, and promoting ATP level in SH-SY5Y cells after MPP^+^ insult, which is correlated with increase of protein levels of ERRα, PPARγ, NRF-1, and NRF-2 (Ye et al., 2016). Taken together, in agreement with our new data from silencing PGC-1α, our data indicate that ERRα, PPARγ, and NRF-1 along with PGC-1α may be involved in the mitochondria protection in MPP^+^-induced cell model of PD.

## Conclusion

In summary, we found that after silencing the PGC-1α gene, SH-SY5Y cells showed increased susceptibility to MPP^+^ and alternation of mitochondrial function, including higher level of H_2_O_2_ and the release of cyt c into cytoplasm. Also, the expression of ERRα, NRF-1, NRF-2 and PPARγ were correspondingly decreased along with PGC-1α silencing. Especially, ERRα showed more correlation among those factors, providing new experimental evidence for understanding the pathogenesis of PD. In addition, studies have found that PGC-1α can be induced by calcium and cAMP signals in common pathways found in most tissues, indicating the possibility of developing a new drug to elevate PGC-1α levels in brain tissue.

## Author Contributions

QY conceived and supervised the study. ES, JW, CC and YW participated in the flow cytometry assay, ELISA assay, immunohistochemistry, western blot analysis, realtime-PCR. CC, YC, DL and WH helped to draft the manuscript; XC also conceived the study. All authors read and approved the final manuscript.

## Conflict of Interest Statement

The authors declare that the research was conducted in the absence of any commercial or financial relationships that could be construed as a potential conflict of interest.
