# Supplementary material for: Mitochondrial Effects of PGC-1alpha Silencing in MPP+ Treated Human SH-SY5Y Neuroblastoma Cells
Source: Front Mol Neurosci. 2017 May 29;10:164. doi: 10.3389/fnmol.2017.00164 (PMC5447087; doi:10.3389/fnmol.2017.00164)
Supplement: Supplementary file 1 [file Table_1.docx]

**Supplemental material 1: antibodies list**

| **Material or reagent name** | **concentration** | **Manufacturer** | **Country** |
| --- | --- | --- | --- |
| NRF-1 (ab34682) antibody | 1:800 | Abcam | Cambridge, MA, USA |
| NRF-2 (ab88746) antibody | 1:1000 | Abcam | Cambridge, MA, USA |
| PPARγ (ab19481) antibody | 1:1000 | Abcam | Cambridge, MA, USA |
| PGC-1α (ST1202-1SET) antibody | 1:1500 | EMD Millipore | Billerica, MA, USA |
| ERRα (Cat. 04-1134) antibody | 1:2500 | EMD Millipore | Billerica, MA, USA |
| TH | 1:100 | Santa Cruz | CA, USA |
| Actin antibody | 1:2000 | Beyotime Company of Biotechnology | Shanghai, China |
| GAPDH antibody | 1:1000 | Beyotime Company of Biotechnology | Shanghai, China |
| goat anti-mouse IgG antibody | 1:2000 | Beyotime Company of Biotechnology | Shanghai, China |
| goat anti-rabbit IgG antibody | 1:2000 | Beyotime Company of Biotechnology | Shanghai, China |
| donkey anti-rabbit IgG | 1:1000 | Invitrogen | Paisley, UK |
