# Supplementary material for: Mitochondrial Effects of PGC-1alpha Silencing in MPP+ Treated Human SH-SY5Y Neuroblastoma Cells
Source: Front Mol Neurosci. 2017 May 29;10:164. doi: 10.3389/fnmol.2017.00164 (PMC5447087; doi:10.3389/fnmol.2017.00164)
Supplement: Supplementary file 2 [file Table_2.docx]

**Supplemental material 1: abbreviations and full name list**

**Abbreviations full name**

PD Parkinson’s disease

PGC-1α peroxisome proliferator-activated receptor γ coactivator 1α

RNAi RNA interference

SN substantia nigra

PPARγ Peroxisome proliferator-activated receptor γ

PRC PGC-1β and PGC-related co-activator

NRF-1 nuclear respiratory factors 1

NRF-2 nuclear respiratory factors 2

ERRα estrogen-related receptor α

cyt c cytochrome c

TFAM mitochondrial transcription factor A

Htt Huntingtin

MPP+ N-methyl-4-phenylpyridinium ion

MPTP 1-methyl-4-phenyl-1,2,3,6-tetrahydropyridine

MTT 3-[4,5-dimethylthiazol-2-yl]-2,5-diphenyl-tetrazolium bromide

DMEM Dulbecco's Modified Eagle’s Medium

FBS fetal bovine serum

MOI multiplicity of infection

CM complete media

OD Optical density

Ad Adenovirus

GFP Green fluorescent protein

BSA bovine serum albumin

PBS phosphate buffered saline

DAB Diaminobenzidine

EDTA Ethylenediaminetetraacetic acid

PMSF phenylmethanesulfonylfluoride

SDS-PAGE sodium dodecyl sulfate-polyacrylamide gel electrophoresis

PVDF polyvinylidene fluoride

ECL enhanced Chemiluminescence

Rh123 Rhodamine 123

HRP horseradish peroxidase

PGC-1 PGC-1α interference RNA 1

PGC-2 PGC-1α interference RNA 2

PGC-3 PGC-1α interference RNA 3

PGC-4 PGC-1α interference RNA 4)

ANOVA one-way analysis of variance

ROS reactive oxygen species

cyt c cytochrome c

NR nuclear receptors

HD Huntington's disease

ALS amyotrophic lateral sclerosis

AD Alzheimer's disease

SiRNA small interfering RNA

IMS intermembrane space

COX cytochrome c oxidase

WT wild-type

SOD1 superoxide dismutase

SOD2 manganese SOD

GPx1 glutathione peroxidase1

LPS lipopolysaccharide

Keap1 kelch-like ECH-associated protein 1

EpRE electrophile response element

TFBM mitochondrial transcription factor B
