# Supplementary material for: Mitochondrial Effects of PGC-1alpha Silencing in MPP+ Treated Human SH-SY5Y Neuroblastoma Cells
Source: Front Mol Neurosci. 2017 May 29;10:164. doi: 10.3389/fnmol.2017.00164 (PMC5447087; doi:10.3389/fnmol.2017.00164)
Supplement: Supplementary file 3 [file Data_Sheet_1.docx]

**Supplemental material 2: the siRNA sequences (yellow) in NM_013261**

TAGTAAGACAGGTGCCTTCAGTTCACTCTCAGTAAGGGGCTGGTTGCCTGCATGAGTGTGTGCTCTGTGTCACTGTGGAT

TGGAGTTGAAAAAGCTTGACTGGCGTCATTCAGGAGCTGGATGGCGTGGGACATGTGCAACCAGGACTCTGAGTCTGTAT

GGAGTGACATCGAGTGTGCTGCTCTGGTTGGTGAAGACCAGCCTCTTTGCCCAGATCTTCCTGAACTTGATCTTTCTGAA

CTAGATGTGAACGACTTGGATACAGACAGCTTTCTGGGTGGACTCAAGTGGTGCAGTGACCAATCAGAAATAATATCCAA

TCAGTACAACAATGAGCCTTCAAACATATTTGAGAAGATAGATGAAGAGAATGAGGCAAACTTGCTAGCAGTCCTCACAG

AGACACTAGACAGTCTCCCTGTGGATGAAGACGGATTGCCCTCATTTGATGCGCTGACAGATGGAGACGTGACCACTGAC

AATGAGGCTAGTCCTTCCTCCATGCCTGACGGCACCCCTCCACCCCAGGAGGCAGAAGAGCCGTCTCTACTTAAGAAGCT

CTTACTGGCACCAGCCAACACTCAGCTAAGTTATAATGAATGCAGTGGTCTCAGTACCCAGAACCATGCAAATCACAATC

ACAGGATCAGAACAAACCCTGCAATTGTTAAGACTGAGAATTCATGGAGCAATAAAGCGAAGAGTATTTGTCAACAGCAA

AAGCCACAAAGACGTCCCTGCTCGGAGCTTCTCAAATATCTGACCACAAACGATGACCCTCCTCACACCAAACCCACAGA

GAACAGAAACAGCAGCAGAGACAAATGCACCTCCAAAAAGAAGTCCCACACACAGTCGCAGTCACAACACTTACAAGCCA

AACCAACAACTTTATCTCTTCCTCTGACCCCAGAGTCACCAAATGACCCCAAGGGTTCCCCATTTGAGAACAAGACTATT

GAACGCACCTTAAGTGTGGAACTCTCTGGAACTGCAGGCCTAACTCCACCCACCACTCCTCCTCATAAAGCCAACCAAGA

TAACCCTTTTAGGGCTTCTCCAAAGCTGAAGTCCTCTTGCAAGACTGTGGTGCCACCACCATCAAAGAAGCCCAGGTACA

GTGAGTCTTCTGGTACACAAGGCAATAACTCCACCAAGAAAGGGCCGGAGCAATCCGAGTTGTATGCACAACTCAGCAAG

TCCTCAGTCCTCACTGGTGGACACGAGGAAAGGAAGACCAAGCGGCCCAGTCTGCGGCTGTTTGGTGACCATGACTATTG

CCAGTCAATTAATTCCAAAACAGAAATACTCATTAATATATCACAGGAGCTCCAAGACTCTAGACAACTAGAAAATAAAG

ATGTCTCCTCTGATTGGCAGGGGCAGATTTGTTCTTCCACAGATTCAGACCAGTGCTACCTGAGAGAGACTTTGGAGGCA

AGCAAGCAGGTCTCTCCTTGCAGCACAAGAAAACAGCTCCAAGACCAGGAAATCCGAGCCGAGCTGAACAAGCACTTCGG

TCATCCCAGTCAAGCTGTTTTTGACGACGAAGCAGACAAGACCGGTGAACTGAGGGACAGTGATTTCAGTAATGAACAAT

TCTCCAAACTACCTATGTTTATAAATTCAGGACTAGCCATGGATGGCCTGTTTGATGACAGCGAAGATGAAAGTGATAAA

CTGAGCTACCCTTGGGATGGCACGCAATCCTATTCATTGTTCAATGTGTCTCCTTCTTGTTCTTCTTTTAACTCTCCATG

TAGAGATTCTGTGTCACCACCCAAATCCTTATTTTCTCAAAGACCCCAAAGGATGCGCTCTCGTTCAAGGTCCTTTTCTC

GACACAGGTCGTGTTCCCGATCACCATATTCCAGGTCAAGATCAAGGTCTCCAGGCAGTAGATCCTCTTCAAGATCCTGC

TATTACTATGAGTCAAGCCACTACAGACACCGCACGCACCGAAATTCTCCCTTGTATGTGAGATCACGTTCAAGATCGCC

CTACAGCCGTCGGCCCAGGTATGACAGCTACGAGGAATATCAGCACGAGAGGCTGAAGAGGGAAGAATATCGCAGAGAGT

ATGAGAAGCGAGAGTCTGAGAGGGCCAAGCAAAGGGAGAGGCAGAGGCAGAAGGCAATTGAAGAGCGCCGTGTGATTTAT

GTCGGTAAAATCAGACCTGACACAACACGGACAGAACTGAGGGACCGTTTTGAAGTTTTTGGTGAAATTGAGGAGTGCAC

AGTAAATCTGCGGGATGATGGAGACAGCTATGGTTTCATTACCTACCGTTATACCTGTGATGCTTTTGCTGCTCTTGAAA

ATGGATACACTTTGCGCAGGTCAAACGAAACTGACTTTGAGCTGTACTTTTGTGGACGCAAGCAATTTTTCAAGTCTAAC

TATGCAGACCTAGATTCAAACTCAGATGACTTTGACCCTGCTTCCACCAAGAGCAAGTATGACTCTCTGGATTTTGATAG

TTTACTGAAAGAAGCTCAGAGAAGCTTGCGCAGGTAACATGTTCCCTAGCTGAGGATGACAGAGGGATGGCGAATACCTC

ATGGGACAGCGCGTCCTTCCCTAAAGACTATTGCAAGTCATACTTAGGAATTTCTCCTACTTTACACTCTCTGTACAAAA

ACAAAACAAAACAACAACAATACAACAAGAACAACAACAACAATAACAACAATGGTTTACATGAACACAGCTGCTGAAGA

GGCAAGAGACAGAATGATATCCAGTAAGCACATGTTTATTCATGGGTGTCAGCTTTGCTTTTCCTGGAGTCTCTTGGTGA

TGGAGTGTGCGTGTGTGCATGTATGTGTGTGTGTATGTATGTGTGTGGTGTGTGTGCTTGGTTTAGGGGAAGTATGTGTG

GGTACATGTGAGGACTGGGGGCACCTGACCAGAATGCGCAAGGGCAAACCATTTCAAATGGCAGCAGTTCCATGAAGACA

CGCTTAAAACCTAGAACTTCAAAATGTTCGTATTCTATTCAAAAGGAAATATATATATATATATATATATATATATATAT

ATATATAAATTAAAAAGGAAAGAAAACTAACAACCAACCAACCAACCAACCAACCACAAACCACCCTAAAATGACAGCCG

CTGATGTCTGGGCATCAGCCTTTGTACTCTGTTTTTTTAAGAAAGTGCAGAATCAACTTGAAGCAAGCTTTCTCTCATAA

CGTAATGATTATATGACAATCCTGAAGAAACCACAGGTTCCATAGAACTAATATCCTGTCTCTCTCTCTCTCTCTCTCTC

TCTCTTTTTTTTTTCTTTTTCCTTTTGCCATGGAATCTGGGTGGGAGAGGATACTGCGGGCACCAGAATGCTAAAGTTTC

CTAACATTTTGAAGTTTCTGTAGTTCATCCTTAATCCTGACACCCATGTAAATGTCCAAAATGTTGATCTTCCACTGCAA

ATTTCAAAAGCCTTGTCAATGGTCAAGCGTGCAGCTTGTTCAGCGGTTCTTTCTGAGGAGCGGACACCGGGTTACATTAC

TAATGAGAGTTGGGTAGAACTCTCTGAGATGTGTTCAGATAGTGTAATTGCTACATTCTCTGATGTAGTTAAGTATTTAC

AGATGTTAAATGGAGTATTTTTATTTTATGTATATACTATACAACAATGTTCTTTTTTGTTACAGCTATGCACTGTAAAT

GCAGCCTTCTTTTCAAAACTGCTAAATTTTTCTTAATCAAGAATATTCAAATGTAATTATGAGGTGAAACAATTATTGTA

CACTAACATATTTAGAAGCTGAACTTACTGCTTATATATATTTGATTGTAAAAACAAAAAGACAGTGTGTGTGTCTGTTG

AGTGCAACAAGAGCAAAATGATGCTTTCCGCACATCCATCCCTTAGGTGAGCTTCAATCTAAGCATCTTGTCAAGAAATA

TCCTAGTCCCCTAAAGGTATTAACCACTTCTGCGATATTTTTCCACATTTTCTTGTCGCTTGTTTTTCTTTGAAGTTTTA

TACACTGGATTTGTTAGGGGAATGAAATTTTCTCATCTAAAATTTTTCTAGAAGATATCATGATTTTATGTAAAGTCTCT

CAATGGGTAACCATTAAGAAATGTTTTTATTTTCTCTATCAACAGTAGTTTTGAAACTAGAAGTCAAAAATCTTTTTAAA

ATGCTGTTTTGTTTTAATTTTTGTGATTTTAATTTGATACAAAATGCTGAGGTAATAATTATAGTATGATTTTTACAATA

ATTAATGTGTGTCTGAAGACTATCTTTGAAGCCAGTATTTCTTTCCCTTGGCAGAGTATGACGATGGTATTTATCTGTAT

TTTTTACAGTTATGCATCCTGTATAAATACTGATATTTCATTCCTTTGTTTACTAAAGAGACATATTTATCAGTTGCAGA

TAGCCTATTTATTATAAATTATGAGATGATGAAAATAATAAAGCCAGTGGAAATTTTCTACCTAGGATGCATGACAATTG

TCAGGTTGGAGTGTAAGTGCTTCATTTGGGAAATTCAGCTTTTGCAGAAGCAGTGTTTCTACTTGCACTAGCATGGCCTC

TGACGTGACCATGGTGTTGTTCTTGATGACATTGCTTCTGCTAAATTTAATAAAAACTTCAGAAAAACCTCCATTTTGAT

CATCAGGATTTCATCTGAGTGTGGAGTCCCTGGAATGGAATTCAGTAACATTTGGAGTGTGTATTCAAGTTTCTAAATTG

AGATTCGATTACTGTTTGGCTGACATGACTTTTCTGGAAGACATGATACACCTACTACTCAATTGTTCTTTTCCTTTCTC

TCGCCCAACACGATCTTGTAAGATGGATTTCACCCCCAGGCCAATGCAGCTAATTTTGATAGCTGCATTCATTTATCACC

AGCATATTGTGTTCTGAGTGAATCCACTGTTTGTCCTGTCGGATGCTTGCTTGATTTTTTGGCTTCTTATTTCTAAGTAG

ATAGAAAGCAATAAAAATACTATGAAATGAAAGAACTTGTTCACAGGTTCTGCGTTACAACAGTAACACATCTTTAATCC

GCCTAATTCTTGTTGTTCTGTAGGTTAAATGCAGGTATTTTAACTGTGTGAACGCCAAACTAAAGTTTACAGTCTTTCTT

TCTGAATTTTGAGTATCTTCTGTTGTAGAATAATAATAAAAAGACTATTAAGAGCAATAAATTATTTTTAAGAAATCGAG

ATTTAGTAAATCCTATTATGTGTTCAAGGACCACATGTGTTCTCTATTTTGCCTTTAAATTTTTGTGAACCAATTTTAAA

TACATTCTCCTTTTTGCCCTGGATTGTTGACATGAGTGGAATACTTGGTTTCTTTTCTTACTTATCAAAAGACAGCACTA

CAGATATCATATTGAGGATTAATTTATCCCCCCTACCCCCAGCCTGACAAATATTGTTACCATGAAGATAGTTTTCCTCA

ATGGACTTCAAATTGCATCTAGAATTAGTGGAGCTTTTGTATCTTCTGCAGACACTGTGGGTAGCCCATCAAAATGTAAG

CTGTGCTCCTCTCATTTTTATTTTTATTTTTTTGGGAGAGAATATTTCAAATGAACACGTGCACCCCATCATCACTGGAG

GCAAATTTCAGCATAGATCTGTAGGATTTTTAGAAGACCGTGGGCCATTGCCTTCATGCCGTGGTAAGTACCACATCTAC

AATTTTGGTAACCGAACTGGTGCTTTAGTAATGTGGATTTTTTTCTTTTTTAAAAGAGATGTAGCAGAATAATTCTTCCA

GTGCAACAAAATCAATTTTTTGCTAAACGACTCCGAGAACAACAGTTGGGCTGTCAACATTCAAAGCAGCAGAGAGGGAA

CTTTGCACTATTGGGGTATGATGTTTGGGTCAGTTGATAAAAGGAAACCTTTTCATGCCTTTAGATGTGAGCTTCCAGTA

GGTAATGATTATGTGTCCTTTCTTGATGGCTGTAATGAGAACTTCAATCACTGTAGTCTAAGACCTGATCTATAGATGAC

CTAGAATAGCCATGTACTATAATGTGATGATTCTAAATTTGTACCTATGTGACAGACATTTTCAATAATGTGAACTGCTG

ATTTGATGGAGCTACTTTAAGATTTGTAGGTGAAAGTGTAATACTGTTGGTTGAACTATGCTGAAGAGGGAAAGTGAGCG

ATTAGTTGAGCCCTTGCCGGGCCTTTTTTCCACCTGCCAATTCTACATGTATTGTTGTGGTTTTATTCATTGTATGAAAA

TTCCTGTGATTTTTTTTAAATGTGCAGTACACATCAGCCTCACTGAGCTAATAAAGGGAAACGAATGTTTCAAATCTAAA

AAAAAAAAAAAAAAA

**SiRNA sequence**

| Marker | gene | Gene ID | TargetSeq | GC% |
| --- | --- | --- | --- | --- |
| pAVT785  (PGC-1) | PPARGC1A | NM_013261 | GCAATAAAGCGAAGAGTAT | 36.9 |
| pAVT786  (PGC-2) | PPARGC1A | NM_013261 | CCACCACTCCTCCTCATAA | 52.6 |
| pAVT787  (PGC-3) | PPARGC1A | NM_013261 | CCGAAATTCTCCCTTGTAT | 42.1 |
| pAVT788  (PGC-3) | PPARGC1A | NM_013261 | GCTATGGTTTCATTACCTA | 36.9 |
| pAVT1  (Ad) | NC |  | TTCTCCGAACGTGTCACGT | 52.6 |

**Virus vector building framework:**

| NO. | 5’ | STEMP | Loop | STEMP | 3’ |
| --- | --- | --- | --- | --- | --- |
| AVT785-1 | Ccgg | GCAATAAAGCGAAGAGTAT | TTCAAGAGA | ATACTCTTCGCTTTATTGC | TTTTTTg |
| AVT785-2 | aattcaaaaaa | GCAATAAAGCGAAGAGTAT | TCTCTTGAA | ATACTCTTCGCTTTATTGC |  |
| AVT786-1 | Ccgg | CCACCACTCCTCCTCATAA | TTCAAGAGA | TTATGAGGAGGAGTGGTGG | TTTTTTg |
| AVT786-2 | aattcaaaaaa | CCACCACTCCTCCTCATAA | TCTCTTGAA | TTATGAGGAGGAGTGGTGG |  |
| AVT787-1 | Ccgg | CCGAAATTCTCCCTTGTAT | TTCAAGAGA | ATACAAGGGAGAATTTCGG | TTTTTTg |
| AVT787-2 | aattcaaaaaa | CCGAAATTCTCCCTTGTAT | TCTCTTGAA | ATACAAGGGAGAATTTCGG |  |
| AVT788-1 | Ccgg | GCTATGGTTTCATTACCTA | TTCAAGAGA | TAGGTAATGAAACCATAGC | TTTTTTg |
| AVT788-2 | aattcaaaaaa | GCTATGGTTTCATTACCTA | TCTCTTGAA | TAGGTAATGAAACCATAGC |  |
| AVT1-1 | Ccgg | TTCTCCGAACGTGTCACGT | TTCAAGAGA | ACGTGACACGTTCGGAGAA | TTTTTg |
| AVT1-2 | aattcaaaaa | TTCTCCGAACGTGTCACGT | TCTCTTGAA | ACGTGACACGTTCGGAGAA |  |
